# Growth performance and hematological responses of silver barb (*Barbonymus gonionotus* bleeker, 1850) fingerlings to dietary blanched moringa (*Moringa oleifera* lam.) leaf meal as a substitute of soybean meal

**DOI:** 10.1016/j.heliyon.2023.e13552

**Published:** 2023-02-07

**Authors:** Farhabun Binte Farhad, Shaharior Hashem, K.M. Shakil Rana, M.A. Salam

**Affiliations:** Department of Aquaculture, Bangladesh Agricultural University, Mymensingh 2202, Bangladesh

**Keywords:** Alternative protein, Moringa leaf meal, Soybean, Growth, Hematology, Silver barb, Stress tolerance

## Abstract

The fastest-growing aquaculture industry relies heavily on animal protein, fishmeal and plant protein to maintain production levels. Therefore, present study was conducted to perceive the effectiveness of blanched moringa (*Moringa oliefera*) leaf meal (MLM) as a replacement of soybean meal in silver barb (*Barbonymus gonionotus*) fingerling diet. Four experimental feeds were prepared replacing soybean meal with MLM at 0, 10, 30, and 50%. Fish were reared for 60 days in 12 hapas installed in three similar size and shaped ponds comprising four treatments each having 3 replications. Fish were fed with the experimental diet at 5% body weight twice daily. Fish growth parameters and length-weight relationship were assessed. To determine their resistance to stressful conditions, the fish were submerged in low pH-5 solution at the conclusion of the experiment. According to the results of the fish growth metrics, the majority of the parameters were comparable and statistically insignificant between the treatments. However, when compared to the control, T_1_ and T_2_ treatments, the T_3_ treatment demonstrated increased survival, PER, and fish production. In addition, other parameters such as percent weight gain, SGR, FCR and FCE were higher in control but statistically similar with T_3_. Besides, the length-weight relationship of silver barb fingerlings fed with all the test diets showed a positive association and isometric growth pattern. With the incremental addition of MLM to the fish diet, the hematological parameters—red blood cells (RBC), white blood cells (WBC), hemoglobin (Hb), and mean corpuscular hemoglobin (MCH) gradually increased. The fish fed the T_3_ diet had the highest recorded stress tolerance (6.50 ± 0.50 min), whereas the fish on the control diet had the lowest (T_0_, 4.77 ± 0.68 min). According to the study, MLM has the potential to replace soybean meal in the diet of silver barb fingerlings to the tune of 50:50 without having an adverse impact on growth. It can enhance fish hematological performance and tolerance for unfavorable environmental conditions as well.

## Highlights

• Blanching can reduce some anti-nutritional compound present in plant origin.
• Blanched *Moringa oleifera* leaf meal (MLM) can be used as an alternative feedstuff.
• Highest inclusion of MLM insignificantly affected the growth of barb fish.
• Better hematological indices and stress tolerance can be reached in 12.5% inclusion.

## 1 Introduction

The aquaculture is one of the most productive, dynamic and fastest growing sector that can contribute animal protein and address malnutrition in developing countries like Bangladesh [1]. Fish and fisheries products are recognized as the healthiest foods on the planet which have less impact on the natural ecosystem [2]. Fishes are considered as vital source of food, nutrition, income, and livelihood options for the rural and coastal communities [3,4]. In Fiscal Year 2019–20, total fish production of Bangladesh was 4.503 million metric tons, where aquaculture contributed 57.38% [5]. The rapid expansion of the aquaculture industry along with the advancement of culture technologies resulted increasing demand for fish feed [6,7]. The nutritional quality of fish feed ingredients are most important than the availability in bulk quantities [8]. Fish feed ingredients are primarily made of plants and animals. Fish meal is made from wild caught fish, which has a significant impact on the wild fish population. On the other side, domesticated birds and animals are contenders for plant-based substances. Due to the lack of conventional feedstuffs essential for sustained aquaculture production, there is a constant need for alternate protein sources [6,9]. Nevertheless, due to the presence of numerous anti-nutritional elements, plant-derived materials are not frequently used in a bulk quantity as fish-feed ingredients [10]. Some authors have examined the effects of MLM on a range of fish species, including Nile tilapia (*Oreochromis niloticus*), Bocourti catfish (*Pangasius bocourti*), African catfish (*Clarias gariepinus***)**, Carpio (*Cyprinus carpio*), and Rohu (*Labeo rohita*) with positive outcomes [[11], [12], [13], [14], [15]].

Moringa is a versatile vegetable tree with many potential uses including nutritional and therapeutic properties. It has enormous potential for generating income and ensuring nutritional security [16]. It is indigenous to north west India, Pakistan, Bangladesh, and Afghanistan that are sub-Himalayan, and is frequently referred to as the "drumstick tree" or the "horseradish tree" [17,18]. According to Gopalakrishnan et al. [19], *M. oleifera* has significant nutrients and anti-nutrients in all of its parts (root, leaf, bark, gum, leaf, fruit, flowers, seed, and seed oil), which have all been used medicinally to treat a variety of illnesses [20]. The ascorbic acid, flavonoids, polyphenols, and carotenoids found in the moringa leaf are possible sources of vitamin A, B, and C, minerals, and amino acids [17,20,21]. Additionally, both in raw and processed forms, plant leaf have crude protein levels between 25.4 and 43.5% [22]. Furthermore, the essential amino acids found in moringa leaves are almost similar to those in soybeans and are above the amino acid pattern of the FAO reference protein [23]. Different parts of *M. oleifera* contain several anti-nutrients such as, phenols, tannins, saponins, phytate, lectins, cyanogenic glucoside, and glucosinolate [22]. By blanching plants in hot water, some anti-nutritional substances including oxalic acid, tannins, HCN, and saponins that are present in plants can be reduced [24,25]. However, blanching can reduce the material's nutritional value when a component is sensitive to high temperature and readily dissolves in water, such as protein and vitamin C [26]. To reduce anti-nutritional content while maintaining the nutritional value of moringa leaves and to preserve the leaves, researchers identified optimal temperature and blanching time restrictions [26,27].

Thai sharpunti (*Barbonymus gonionotus* Bleeker, 1850), belongs to the Cyprinidae family, is frequently cultivated species in Bangladesh. It arrived from Thailand for the first time in the country in 1977 [28]. Due to its superior palatability, high output potential, and market demand among all the exotic fish species of Bangladesh, it is regarded as one of the most ideal aquaculture species [29]. This species is easily farmed in seasonal ponds, ditches, and roadside canals and can withstand shallow, murky waters and reach table size in three to four months [30,31].

In toxicological research, it is believed that understanding fish hematological features is essential to comprehending fish immunology. The hematological parameters are readily available tools that fish biologists and researchers use all over the world [32]. They are crucial for assessing the health status, physiological conditions, disease, as well as the impact of feed and other environmental parameters in cultured fish [[33], [34], [35]]. Due to dietary composition, metabolic adaptability, and variations in fish activity, the parameters can fluctuate [36]. Additionally, both internal and external variables can affect the hematological parameters [37]. Blood parameters, on the other hand, are helpful indicators of physiological changes in intensively farmed fishes and can offer crucial information for the diagnosis and forecast of disease [38]. Therefore, the aim of the present study is to evaluate the effectiveness of blanched MLM as a substitute of soybean meal by assessing the growth performance and hematological indices of silver barb fingerlings.

## 2 Materials and methods

### 2.1 Experimental site

The experiment was carried out in three (3) similar size and depth individual ponds of 2 decimals at the southern side of the Faculty of Fisheries, Bangladesh Agricultural University, Mymensingh, Bangladesh. The duration of the experiment was sixty (60) days from 20th September to November 18, 2019.

### 2.2 Experimental fish

Healthy and vigorous silver barb, *Barbonymus gonionotus* (approximately 250) fingerlings were collected from Bangladesh Fisheries Research Institute (BFRI), Mymensingh, Bangladesh.

### 2.3 Experimental design

Fish rearing hapas (2 m × 1 m × 1 m) were prepared using mosquito nets and installed in three individual ponds (each pond with 4 hapas) with the help of bamboo and tied with rope. Purposive random sampling methodology was adopted to allocate the treatments among the hapas in the three ponds. In the treatments progressive replacement of soybean meal with MLM at 0, 10, 30 and 50% were done and denoted as T_0_, T_1_, T_2_, and T_3_ respectively. Each treatment had three replications. The collected fingerlings were stocked into two experimental hapas for fifteen days to acclimatize with the environmental condition and fed with a commercial diet (Mega fish feed Ltd. Bangladesh) containing 30% protein, twice daily. Followed by acclimatization, the fingerlings were distributed randomly among four treatments in three ponds at a stocking density of 9 fingerlings per m^3^ with an average initial length and weight of 7.41 ± 0.53 cm and 5.77 ± 0.26 g, respectively. Prior to the commencement of the experiment, the fingerlings were starve for 24 h to empty their stomach for actual weight and accepting the new diet following day. The fish was fed twice a day (9.00 a.m. and 5.00 p.m.) at the rate of 5% of their body weight. The fish was sampled randomly with a scope net once in 15 days interval. Additionally, several water quality parameters were evaluated using sera aqua-test kits near the edges of the ponds.

### 2.4 Preparation of moringa leaf meal (MLM)

Fresh moringa leaves were collected from trees located near the pond edges at the southern side of the Department of Aquaculture, BAU, Mymensingh. After collection, the leaves were separated from the branches and then blanched using boiling water for 7.5 min and immediately dripped into ice water to stop the cooking process. The blanched leaves were then kept overnight in room condition to remove excess water. The following day, the leaves were put in a homemade drier at 40 °C temperature until completely drying. Thereafter, the leaves were ground into fine powder using a blender. The powder was then sieved to separate the bigger parts of the leaves and kept in the fridge (at 4 °C temp.). The proximate composition of MLM was assessed in Fish Nutrition Laboratory (BAU) following the standard protocol [39].

### 2.5 Ingredients selection and diet formulation

The ingredients chosen and purchased for diet formulation were fish meal, soybean meal, rice polish, wheat bran, wheat flour, vitamin-mineral premix, molasses along with MLM. Prior to feed formulation, the proximate composition of all the ingredients was determined. The diets were prepared using Pearson’s square method having 30% protein level (Table 1). The MLM was incorporated in the diet at an inclusion level of 0, 2.5, 7.5, and 12.5 g to replace 0, 10, 30, and 50% of soybean meal, respectively. The feed components were thoroughly mixed with wheat flour acting as a binder before being fed through a pelletizer that produced 2 mm-diameter pellets. The pelleted feeds were Sun-dried for 2 days and stored in the fridge in air tied containers (at 4 °C temp.). The proximate composition of the prepared diets was also analyzed (Table 2).

**Table 1:** Percentage inclusion level of ingredients for diet preparation.

	Treatments			
Ingredients	T_0_	T_1_	T_2_	T_3_
**Fish meal**	34	34	34	34
**Soybean meal**	25	22.5	17.5	12.5
**MLM**	0	2.5	7.5	12.5
**Rice polish**	13	24	29	33
**Wheat bran**	16	10	5	3
**Starch-wheat flour**	10	5	5	3
**Vit. Min. Premix**	1	1	1	1
**Molasses**	1	1	1	1
**Total**	100	100	100	100

**Table 2:** Proximate composition of MLM and fish feed ingredients.

Nutrients Composition (%)	MLM	Fish meal	Soybean meal	Rice polish	Wheat bran	Wheat flour	Vitamin-mineral premix (per 2.5 Kg)
Moisture	9.32	7.2	15.51	12.97	13.62	13.11	**Vitamins:** A 10^7^ iμ, D3 25 × 10^5^ iμ, E 12000 mg, K 4000 mg, B1 1000 mg, B2 2000 mg, B6 2000 mg, B12 10^4^ mcg, Nicotinic acid 25000 mg, Pantothenic acid 1000 mg, Folic acid 1000 mg, Biotin 50000 mcg **Minerals:** Iron 31500 mg, Copper 6000 mg, Manganese 50000 mg, Cobalt 245 mg, Zinc 50000 mg, Iodine 600 mg
Crude lipid	2.45	10.60	8.20	11.56	3.90	2.21	
Crude protein	24.34	52.71	35.58	12.34	10.68	8.47	
Crude fibre	19.65	2.51	7.40	7.64	8.40	2.24	
Ash	7.23	13.73	7.29	8.3	1.51	1.22	
NFE	37.01	13.25	26.02	47.19	61.89	72.75	

### 2.6 Evaluation of fish growth parameters

Fish growth parameters were determined and calculated as follows-

Weightgain,WG(g)=finalweight(g)–initialweight(g)

$$Percent\;weight\;gain,PWG\;\left(\%\right)=\frac{weight\;gain\;\left(g\right)}{initial\;weight\;\left(g\right)}\times 100$$

$$Specific\;growth\;rate,SGR\;\left(\%\;per\;day\right)=\frac{\log\left[final\;weight\;\left(g\right)\right]–\;\log\left[initial\;weight\;\left(g\right)\right]}{no.\;of\;days}\times 100$$

$$Food\;conversion\;ratio,FCR=\frac{total\;feed\;intake\;\left(g\right)}{total\;wet\;weight\;gained\;\left(g\right)}$$

$$Feed\;conversion\;efficiency,FCE=\frac{Biomass\;\left(g\right)}{Total\;feed\;intake\;\left(g\right)}$$

$$Protein\;efficiency\;ratio,PER=\frac{weight\;gained\;\left(g\right)}{crude\;protein\;fed\;\left(g\right)}$$

$$Survival\;rate,SR\;\left(\%\right)=\frac{\left(Initial\;number\;of\;fish\;stocked\;–\;mortality\right)}{Initial\;number\;of\;fish\;}\times 100$$

### 2.7 Estimation of length-weight relationship

The length-weight relationship of the experimental fish can be represented by the cubic or power curve equation given by Le Cren [40]: W = qL^b^ where, W is the weight of fish (g), L is observed total length (cm), ‘q’ is the regression intercept and ‘b’ is the regression slope which is close to 3 in isometric growth. The logarithmic transformation of the above formula is-

ln W = ln q + b (ln L)

This equation is the same form as linear equation.

Y = a + bx

### 2.8 Assessment of hematological parameters

During the experimental period, several hematological parameters such as red blood cells (RBCs), white blood cells (WBCs), hemoglobin (Hb), and mean corpuscular hemoglobin (MCH) were measured. The blood was collected through a micropipette cutting caudal peduncle of the experimental fish. To prevent blood clotting hematocrit tube was used to draw the blood [41]. The collected samples were kept in Eppendorf tubes and stored in a refrigerator (at 4 °C temp.). RBCs and WBCs count were done with the help of Neubauer Hemocytometer under light microscope following the process of Hesser [41]. Hb concentration was measured using cyan-methemoglobin following the method described by Blaxhall and Daisley [42]. RBCs, WBCs, and MCH were estimated through the following formula [43]-

$$RBC\;count=\frac{Number\;of\;large\;square\;cell\;\left(5\right)\times Dilution\;factor\;\left(200\right)\times Counting\;factor\;\left(4000\right)}{Number\;of\;small\;squares\;counted\;\left(80\right)}$$

$$WBC\;count=\frac{Number\;of\;large\;square\;cell\;\left(1\right)\times Dilution\;factor\;\left(40\right)}{Volume\;factor\;\left(0.1\right)}$$

$$MCH\;\left(pg\right)=\frac{Hb\times 10}{RBC}$$

### 2.9 Sampling water quality parameters

Several water quality parameters such as temperature, dissolved oxygen (DO), pH, ammonia (NH_3_), TDS, phosphate (PO_4_), and EC were assessed and recorded. The temperature was measured using a hand held thermometer. Dissolve oxygen, pH, NH_3_, and PO_4_ were estimated through Sera test kits, sera GmbH, D 52518 (made in Germany) following appropriate procedures at fifteen days interval.

### 2.10 Measurement of low pH-5 stress

To gauge the studied fish's ability to withstand a hostile environment and determine whether MLM had any impact on that pH, the fish were observed under low pH-5 stressful condition. After termination of the experiment, fishes were kept in a separate bucket containing 10 L underground water and pH was reduced to 5.0 pouring vinegar in the water drop by drop. The other water quality parameters such as temperature, DO and NH_4_^+^ remained in ambient condition. The fish were observed until they become unconscious and the time required to become fainted was recorded.

### 2.11 Statistical analysis

Values of all variables were subjected to one-way analysis of variance (ANOVA) and represented as mean ± standard deviation (SD). The significance of the difference between means was carried out by Duncan Multiple Range Test at a significant level of p < 0.05. All computation was performed using the statistical package IBM SPSS Statistics 25.0.

## 3 Results

### 3.1 Nutritional composition and feed quality

The crude protein and crude lipid contents of MLM were 24.34 and 2.45%, respectively. The other components such as moisture, crude fibre, ash, and nitrogen free extract (NFE) were estimated as 9.32, 19.65, 7.23, and 37.01% respectively. The proximate composition of blanched MLM and other feed ingredients are represented in Table 2, and experimental diets in Table 3. The crude protein contents in the formulated feeds chronologically decreased with the increase of MLM were recorded in all the treatments, whereas the lowest value was obtained in T_3_ treatment. In addition, the highest crude lipid content was reported in control (T_0_) while the lowest was detected in the T_2_. At the same time, crude fibre content was recorded higher in T_3_ and a lower value was identified in T_0_.

**Table 3:** Proximate composition of diets prepared with different levels of MLM.

	Treatments			
Proximate composition (%)	T_0_	T_1_	T_2_	T_3_
**Moisture**	12.03	11.48	12.13	11.69
**Crude Protein**	31.13	30.89	29.03	27.15
**Crude Lipid**	6.88	6.32	5.77	5.9
**Crude Fibre**	4.46	5.3	5.4	6.4
**Ash**	8.31	9.07	8.53	9.35
**NFE**	37.19	36.94	39.14	39.51

### 3.2 Fish growth performance

In the present investigation, the most of the fish growth parameters didn't demonstrate any statistically significant changes (p > 0.05) between the treatments (Table 4). However, in the majority of instances, treatment T_1_, which included 10% MLM in the diet, displayed considerably lower values (p < 0.05) than the other treatments. Moreover, when compared to the T_1_ treatment, the control (T_0_) showed significantly higher results for PWG, SGR, PER, and FCE. On the other hand, there was no significant difference between T_0_ and T_3_'s growth metrics, with the exception of PER (1.37 ± 0.01 and 1.53 ± 0.07), where T_3_ demonstrated significantly higher result. Although, T_0_ treatment ingested a higher amount of feed than the other treatments, but the difference was statistically similar. The T_0_ had the lowest FCR when compared to other treatments, which were only statistically significant with T_1_ but not with T_2_ and T_3_. Furthermore, treatment T_3_ had the highest fish production and survival, with MLM replacing 50% of the soyabean meal in the fish diet compared to the other groups (Table 4).

**Table 4:** Growth performance, nutrient utilization, survival rate and production of silver barb fingerlings fed varying levels of MLM-based diet substituted with soybean meal for a period of 60 days.

Treatments							
Growth parameters	T_0_	T_1_	T_2_	T_3_	F value	P value	Level of sig.
**Initial weight (g)**	5.84 ± 0.19	5.75 ± .29	5.47 ± .26	5.76 ± 0.28	1.205	0.368	NS
**Final weight (g)**	20.85 ± 1.12	18.70 ± 1.06	18.98 ± 1.28	20.33 ± 0.97	2.507	0.133	NS
**Weight gain (g)**	15.01 ± 0.93^a^	12.95 ± 0.87^b^	13.51 ± 1.03^ab^	14.57 ± 0.69^ab^	3.382	0.075	NS
**PWG (%)**	256.68 ± 8.11^a^	225.15 ± 4.29^b^	246.78 ± 8.60^a^	253.16 ± 9.34^a^	14.902	0.001	**
**Feed intake (g)**	35.29 ± 2.19	34.30 ± 2.31	32.95 ± 1.94	35.11 ± 2.93	0.605	0.630	NS
**SGR (%/day)**	0.92 ± 0.02^a^	0.85 ± 0.01^b^	0.90 ± 0.02^a^	0.91 ± 0.01^a^	16.267	0.001	**
**FCR**	2.35 ± 0.01^b^	2.65 ± .03^a^	2.44 ± 0.05^b^	2.41 ± 0.11^b^	12.916	0.002	**
**Protein intake**	10.98 ± 0.68^a^	10.59 ± 0.71^ab^	9.56 ± 0.56^b^	9.53 ± 0.80^b^	3.348	0.076	NS
**PER**	1.37 ± 0.01^b^	1.22 ± 0.01^c^	1.41 ± 0.03^b^	1.53 ± 0.07^a^	37.048	0.000	**
**FCE**	0.43 ± 0.01^a^	0.38 ± 0.01^b^	0.41 ± 0.01^a^	0.42 ± 0.02^a^	14.083	0.001	**
**Survival rate (%)**	94.44 ± 5.56^ab^	87.04 ± 3.21^b^	94.44 ± 5.56^ab^	96.29 ± 3.21^a^	4.606	0.037	*
**Production (kg/ha)**	1176.30 ± 99.12^ab^	1098.13 ± 41.04^b^	1057.82 ± 82.56^b^	1261.78 ± 40.43^a^	5.530	0.024	*

Values given in bracket are standard deviation. The values in the same row having similar letter (s) do not differ significantly otherwise differ significantly (p < 0.05) as per DMRT (Duncan Multiple Range Test). NS= Not significant, * = significant in 5%, ** = significant in 1% significance level.

### 3.3 Length-weight relationship

**Fig. 1:**
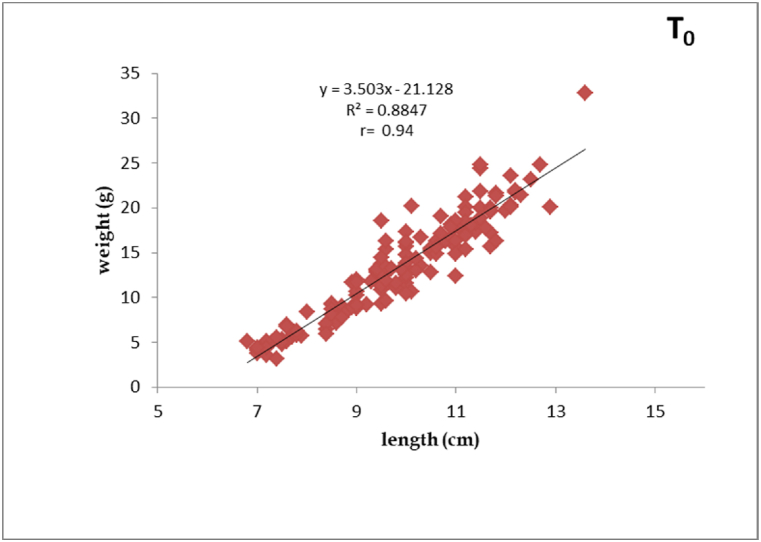
Length-weight relationship of silver barb fingerling fed T_0_ diet.

**Fig. 2:**
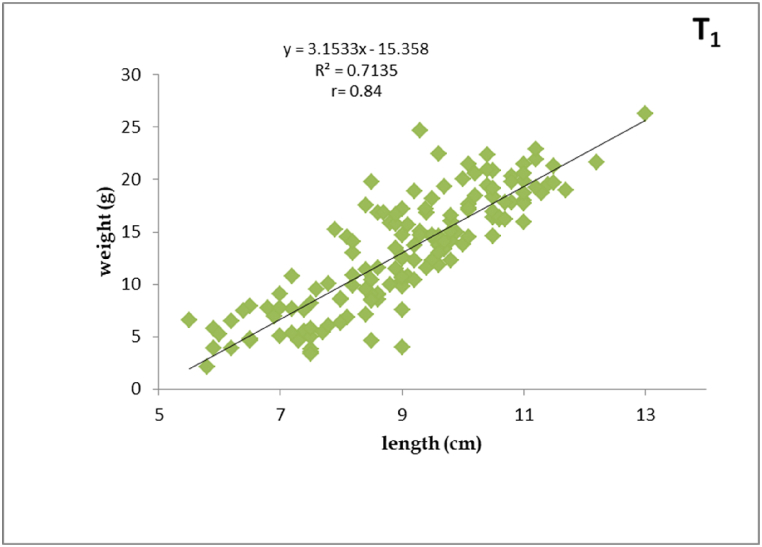
Length-weight relationship of silver barb fingerling fed T_1_ diet.

**Fig. 3:**
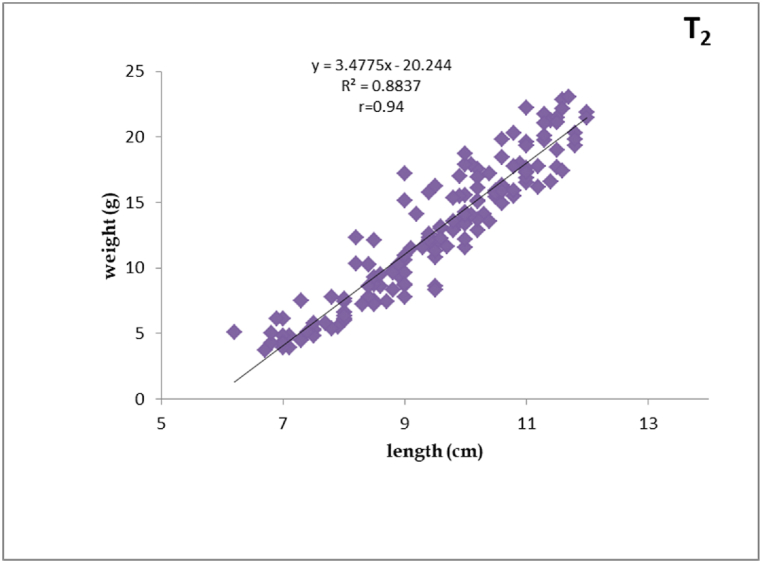
Length-weight relationship of silver barb fingerling fed T_2_ diet.

**Fig. 4:**
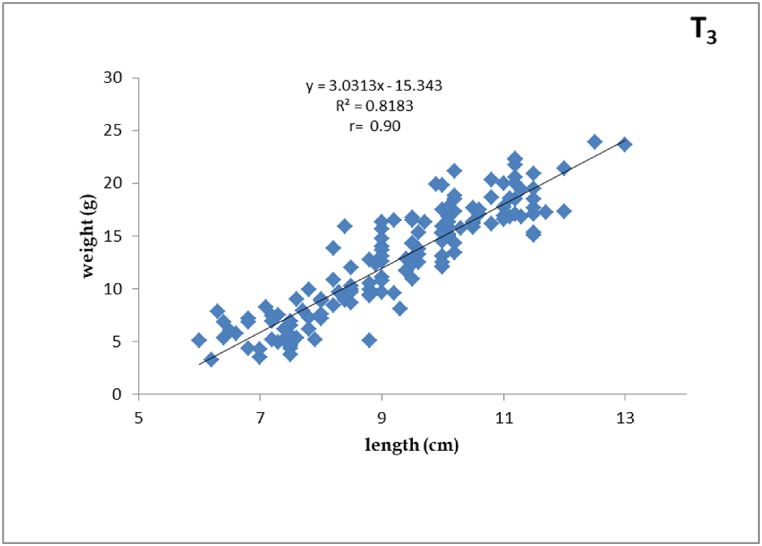
Length-weight relationship of silver barb fingerling fed T_3_ diet.

The interpretation made from the regression equation explained that positive correlation (r = 0.94, 0.84, 0.94, and 0.90) existed between the length and weight of silver barb fingerlings feeding T_0_, T_1_, T_2_ and T_3_ diets, respectively. The regression exponent of ‘b’ values reported from all the treatments were more than 3, indicating that there exhibited positive isometric growth pattern among the experimental fish. The regression graphs of length-weight correlation were presented in Fig. 1, Fig. 2, Fig. 3, Fig. 4.

### 3.4 Hematological parameters

In the present study, the hematological parameters such as RBCs, WBCs, Hb, and MCH were measured (Table 5). The RBCs recorded in the experiment differed significantly (p < 0.05) among all the treatments. The highest RBC was observed in T_3_ (2.56 ± 0.23), while the lowest was detected in T_0_ (1.66 ± 0.34) treatment. The WBCs was also found higher in T_3_ (323.73 ± 4.88), whereas the lowest was recorded in T_2_ (297.00 ± 7.32). The WBCs values were significantly different (p < 0.05) among the treatments. The Hb contents of fish blood were significantly (p < 0.05) increased with increasing dietary incorporation of MLM in the treatments. In the present experiment, the MCH was statistically similar in all the treatments.

**Table 5:** Hematological parameters of silver barb fingerlings fed varying levels of MLM-based diet substituted with soybean meal for a period of 60 days.

Treatments							
**Blood parameters**	**T**_**0**_	**T**_**1**_	**T**_**2**_	**T**_**3**_	F value	P value	Level of sig.
**RBC(×106mm-3)**	1.66 ± 0.34^b^	2.07 ± 0.25^ab^	2.31 ± 0.26^a^	2.56 ± 0.23^a^	5.886	0.020	*
**WBC (×103mm-3)**	304.67 ± 6.46^bc^	312.60 ± 4.65^ab^	297.00 ± 7.32^c^	323.73 ± 4.88^a^	11.14	0.003	**
**Hb (g/100 ml)**	4.40 ± 0.46^c^	4.83 ± 0.32^bc^	5.23 ± 0.21^ab^	5.73 ± 0.45^a^	6.929	0.013	*
**MCH (pg)**	27.02 ± 4.70	23.55 ± 3.30	22.93 ± 3.29	22.46 ± 1.77	1.094	0.406	NS

Values given in bracket are standard deviation. The values in the same row having similar letter (s) do not differ significantly otherwise differ significantly (p < 0.05) as per DMRT (Duncan Multiple Range Test). NS= Not significant, * = significant in 5%, ** = significant in 1% significance level.

### 3.5 Water quality parameters and low pH-5 stress test

In the present experiment, the water quality parameters such as Temperature, DO, pH, NH_3_, PO_4_, TDS, and EC didn’t significantly differ (p > 0.05) among the treatments (Table 6). The water quality parameters were within the suitable range. However, ammonia showed increasing trend at the termination of the experiment.

**Table 6:** Water quality parameters of silver barb fingerlings fed varying levels MLM-based diet substituting with soybean meal for a period of 60 days.

Treatments				
Water quality parameters	T_0_	T_1_	T_2_	T_3_
**Temperature (⁰C)**	28.13 ± 0.20	27.97 ± 0.22	28.01 ± 0.28	28.19 ± 0.53
**Dissolve oxygen (ppm)**	5.58 ± 0.16	5.53 ± 0.15	5.51 ± 0.17	5.40 ± 0.28
pH	7.54 ± 0.04	7.57 ± 0.07	7.51 ± 0.01	7.49 ± 0.02
**NH**_**3**_**(ppm)**	0.30 ± 0.09	0.50 ± 0.26	0.47 ± 0.26	0.43 ± 0.06
**PO**_**4**_**(ppm)**	0.92 ± 0.14	0.83 ± 0.14	1.00 ± 0.25	1.17 ± 0.14
**TDS (ppm)**	128.17 ± 2.02	130.33 ± 8.81	130.33 ± 6.11	134.00 ± 3.61
**EC (⁰F)**	89.00 ± 0.52	88.70 ± 0.00	89.00 ± 0.52	89.12 ± 0.45

**Fig. 5:**
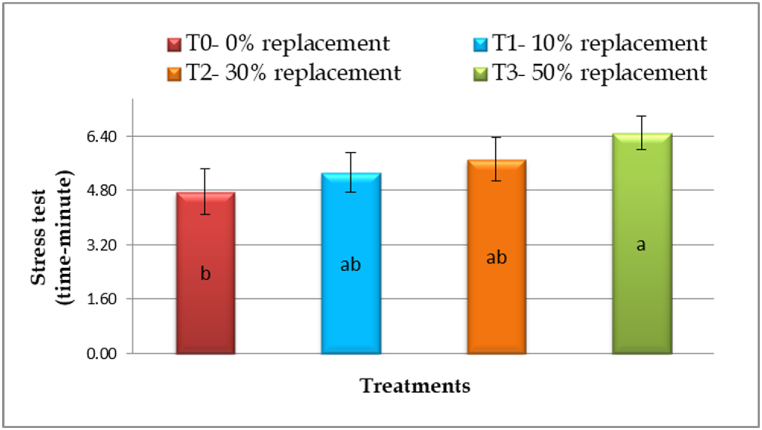
Stress test (minute ± SD) by pH 5 of silver barb fingerling feeding varying level of *M. oleifera* leaf meal based diet (vertical bar of each treatment represents standard deviation).

The low pH 5 stress test revealed significant (p < 0.05) variations across all the treatments. The T_0_ treatment had the lowest tolerance to low pH (4.77 ± 0.68 min), whereas the T_3_ treatment had the highest (6.50 0.50 min). Additionally, T_1_ and T_2_ treatments indicated an intermediate time range of tolerance on low pH, 5.33 ± 0.58 and 5.73 ± 0.64 min, respectively (Fig. 5).

## 4 Discussion

The goal of the current experiment was to find a novel, conveniently accessible, nutrient-rich, mineral- and vitamin-dense MLM to solve the problem of fish feed. In the current study, Thai silver barb fingerlings were fed diets containing different levels of MLM. The growth performance of the fish fed MLM based diets revealed that the mean final weight and mean weight gain were statistically similar to the control. The outcomes are similar to the results reported by Puycha et al. [12] and Adeshina et al. [14]. In contrast, the values attained in the present investigation were lower compared to the findings of Adeshina et al. [14] where they fed MLM based diet to the common carp (*Cyprinus carpio*) juveniles. However, this variation may be attributed to the difference of protein levels in the formulated diets.

The SGR of T_2_ and T_3_ treatments was statistically comparable to that of the control, showing that the growth trend was satisfactory with higher level of MLM inclusion in the diets. Puycha et al. [12] reported improved SGR while feeding MLM based feeds comprising of 100 g/kg feed for 60 days to the Bocourti catfish. The present findings showed that feed consumption did not significantly differ between the treatments, indicating that the fishes' appetites not influenced by the taste of the diets containing different levels of MLM. By contrast, Afaung et al. [44]. Fish fed raw moringa leaves or methanol extracts reduced the feed intake. The variations could be attributed from the use of leaves that have undergone various processing, such as raw, freeze-drying, sun-drying, boiling, and blanching, etc., or from leaf extracts. Indriasari et al. [26]. Blanching moringa leaves at for 7.5 min can reduce the saponins content to the lowest possible level (3.9%), while maintaining the protein level at 25.08%. The blanching method may help to minimize the anti-metabolites in plant-based diets (phenol, tannins, phytate, and saponins), as well as it can lower the oxalate, phytate, and trypsin inhibitor by 20.7, 39.8, and 60.6%, respectively [45]. Moreover, the leaves also exhibited higher carotenoid content, such as TRP, and WAC [46].

The crude protein content was comparatively lower in T_3_ diet than the control, while the protein efficiency ratio was found to be greater in T_3_ treatment, indicating that the source of protein may have improved the ability of fish to utilize critical amino acids. Similar results of protein variation in red tilapia (*Oreochromis* sp.) fed with fermented moringa leaves based feed reported by Helmiati et al. [47]. In addition, Adeshina et al. [14] reported the similar result where moringa based feed showed a higher protein efficiency ratio than the control. Although the protein is the single most important component of fish feed [48], however, Ozovehe and Nzeh [49]other nutrient components present in MLM have the ability to supersede the proteins’ role.

The FCR value was lower in control than T_2_ and T_3_ treatments but without having significant differences. Similar result was reported by Chen et al. [11] with Nile tilapia fed 10% dietary inclusion of MLM. The research conducted by Afuang et al. [44] suggested that 33% solvent-extracted MLM could be incorporated in tilapia diets without having a detrimental effects on growth performances. Additionally, Manuel et al. [50] 30% blanched MLM in fish diet can serve the purposes. The disparity in findings between studies may be due to the various methods MLM is processed, how it is substituted with other ingredients, how much MLM is included in the diet, or any number of other variables that affect fish development and performances. In contrast, higher replacement of raw MLM with fish meal showed poor growth performances [49,[51], [52], [53]], which may be related to the anti-nutritional factors present in the moringa leaf [54].

The length-weight relationship offers information on a species growth and well-being [55,56]. The silver barb fingerlings in the current study displayed isometric growth throughout the experimental period when fed the tested diets. Similarly, Shahabuddin et al. [57] reported isometric (b = 3) exponential growth of Nile tilapia in a recirculating aquaculture system fed with different levels of red alga (*Pyropia spheroplasts*) based diets. In addition, Ngodhe and Owuor [58] positive allometric growth for both wild and cage reared Nile tilapia.

Although several studies reported that increasing level of MLM in fish diet reduces the growth performances of fish, however, boost up the immunological strength [59]. The results of the hematological assessment of the current study showed that the RBCs and Hb content increased with the increase of MLM in the diets. The findings suggested that the tested fish may have stronger immune responses that enhanced the blood properties. Moreover, the higher concentration of micronutrients in the MLM may have contributed the higher Hb contents in the fish. According to Ayoola et al. [60], *Clarias gariepinus* had more RBCs and hemoglobin fed MLM based diet compared to the control. In the current investigation, the Hb, RBCs, and WBCs content grew progressively with the increase of MLM levels in the fish diets except T_2_ treatment, which is supported by the findings of Ezekiel et al. [13], Dienye and Olumuji [51], Ayoola et al. [60].

The robustness of fishes is the indicator of their ability to adjust and adapt to any stressful conditions [61]. The higher tolerance was achieved in fish fed T_3_ diet in low pH of 5 stress test, this could be attributed the fish's higher WBC content Rana et al. [62] reported that the lowest tolerance to low pH stressors (pH 3) was 8 min fish fed control diet, while the highest tolerance was 17 min with 20% jute leaf meal based experimental diet. In the present study, the higher tolerance of the fish fed MLM based diet may be the presence of antioxidants and nutritional value of MLM which is supported by previous findings.

## 5 Conclusion

In the present study, the results showed that the control diet performed better growth performance than MLM based diet, however, T_2_ and T_3_ experimental diets were statistically similar with the control. In contrast, blood parameters RBC, WBC and Hb were much higher in T_2_ and T_3_ treatments and resistance to low pH were significantly higher in all the experimental diets means MLM had positive effects on Thai silver barb wellbeing and robustness than the control. Therefore, the fish culturists can supplement moringa leaves in their fish diet as it is easily available and can be introduced an alternative source of protein.

## Author contribution statement

Farhabun Binte Farhad: Performed the experiments; Analyzed and interpreted the data; Wrote the paper.

Shaharior Hashem: Conceived and designed the experiments; Analyzed and interpreted the data.

K. M. Shakil Rana: Analyzed and interpreted the data.

M. A. Salam: Conceived and designed the experiments; Analyzed and interpreted the data; Contributed reagents, materials, analysis tools or data.

## Funding statement

Nil.

## Data availability statement

Data included in article/supp. material/referenced in article.

## Declaration of interest’s statement

The authors declare no competing interests.
